# Investigating the Effect of Growth Phase on the Surface-Layer Associated Proteome of *Lactobacillus acidophilus* Using Quantitative Proteomics

**DOI:** 10.3389/fmicb.2017.02174

**Published:** 2017-11-08

**Authors:** Courtney Klotz, Sarah O'Flaherty, Yong Jun Goh, Rodolphe Barrangou

**Affiliations:** ^1^Genomic Sciences Graduate Program, North Carolina State University, Raleigh, NC, United States; ^2^Department of Food, Bioprocessing and Nutrition Sciences, North Carolina State University, Raleigh, NC, United States

**Keywords:** *Lactobacillus*, probiotic, cell surface, S-layer, quantitative proteomics

## Abstract

Bacterial surface-layers (S-layers) are semi-porous crystalline arrays that self-assemble to form the outermost layer of some cell envelopes. S-layers have been shown to act as scaffolding structures for the display of auxiliary proteins externally. These S-layer associated proteins have recently gained attention in probiotics due to their direct physical contact with the intestinal mucosa and potential role in cell proliferation, adhesion, and immunomodulation. A number of studies have attempted to catalog the S-layer associated proteome of *Lactobacillus acidophilus* NCFM under a single condition. However, due to the versatility of the cell surface, we chose to employ a multiplexing-based approach with the intention of accurately contrasting multiple conditions. In this study, a previously described lithium chloride isolation protocol was used to release proteins bound to the *L. acidophilus* S-layer during logarithmic and early stationary growth phases. Protein quantification values were obtained via TMT (tandem mass tag) labeling combined with a triple-stage mass spectrometry (MS3) method. Results showed significant growth stage-dependent alterations to the surface-associated proteome while simultaneously highlighting the sensitivity and reproducibility of the technology. Thus, this study establishes a framework for quantifying condition-dependent changes to cell surface proteins that can easily be applied to other S-layer forming bacteria.

## Introduction

The FAO/WHO defines probiotics as “live microorganisms which when administered in adequate amounts confer a health benefit on the host” (Hill et al., 2014). Contributions to host health occur via three proposed mechanisms: competitive exclusion of pathogenic bacteria, enhancement of epithelial barrier function, and modulation of the immune system (Bron et al., 2012). Lactic acid bacteria (LAB) are Gram-positive, non-pathogenic microorganisms characterized by their propensity to metabolize carbohydrates into lactic acid (Pot et al., 1994). Historically, they have been exploited for food and feed fermentations, but more recently have gained attention for the health-promoting properties of some strains (Lebeer et al., 2008; Kleerebezem and Vaughan, 2009). In fact, the incorporation of lactobacilli and bifidobacteria into food and dietary supplements has generated a multimillion dollar business (Kleerebezem and Vaughan, 2009).

Beneficial effects of some *Lactobacillus* strains have been linked with specific surface molecules or protein and metabolite secretions that directly interact with the host (Lebeer et al., 2008). Surface-layers (S-layers) have been detected on many but not all *Lactobacillus* species (Hynönen and Palva, 2013). The bacterial S-layer is a two-dimensional self-assembling crystalline array composed of numerous identical non-covalently bound S-layer proteins (Slps) that form the outermost coating of certain cell envelopes (Fagan and Fairweather, 2014; Sleytr et al., 2014). S-layers have been characterized for their role in a number of processes including maintaining cell shape, acting as molecular sieves, serving as binding sites, and mediating bacterial adhesion (Sleytr et al., 2014). They may also act as a scaffold for the external display of additional proteins or glycoproteins. Supplemental functionality will depend on which proteins the S-layer is presenting (Fagan and Fairweather, 2014). Despite the significance of extracellular proteins in probiotic efficacy, the function of most is still unknown or poorly characterized (Kleerebezem et al., 2010).

*Lactobacillus acidophilus* NCFM is an S-layer forming organism that has been incorporated into food and dietary supplements for over 40 years. Its fully sequenced genome has proven vital in elucidating the underlying molecular mechanisms responsible for probiotic efficacy (Sanders and Klaenhammer, 2001; Altermann et al., 2005). Two recent studies have attempted to catalog the S-layer associated proteome of *L. acidophilus* NCFM via LC-MS/MS (Johnson et al., 2013) and 2-DE in conjunction with MALDI-TOF MS (Celebioglu and Svensson, 2017). However, because the bacterial cell surface is dynamic, there is a need for a more reproducible quantitative technology capable of contrasting multiple conditions.

In mass spectrometry (MS)-based quantification methods, inconsistent ion selection for fragmentation between runs or low quality spectra may result in missing observations, subsequently affecting identification and quantification (Rauniyar and Yates, 2014). However, the introduction of tandem mass spectrometry (MS2) in conjunction with isobaric labels such as tandem mass tags (TMT), has greatly increased the depth of MS-based protein quantification by permitting multiplexing (Thompson et al., 2003). Use of this technology eliminates between run variability and has proven to be a powerful tool for monitoring temporal expression patterns of proteins (Rauniyar and Yates, 2014). Conversely, the accuracy and precision of MS2 can suffer due to co-selection of contaminants with target ions, resulting in an underestimation of fold change (Ow et al., 2009; Christoforou and Lilley, 2012). However, an additional isolation and fragmentation step (MS3) has been shown to overcome this issue, thus eliminating the interference effect (Ting et al., 2011).

In the present study, a previously described lithium chloride (LiCl) isolation protocol (Johnson et al., 2013) was used to release proteins non-covalently bound to the *L. acidophilus* NCFM S-layer during logarithmic and early stationary growth phases. To avoid potential ratio compression effects, protein quantification values were obtained using TMT-based reporter ions in conjunction with a synchronous precursor selection (SPS)-based MS3 technology. As far as we know, this is the first time that a multiplexing proteomic technology has been applied specifically to investigate the probiotic cell surface proteome. Through this approach, we demonstrated significant growth-stage-induced alterations to the *L. acidophilus* non-covalent exoproteome and identified several candidate proteins for functional characterization and cell surface engineering. More importantly, this research establishes a framework for examining condition-dependent cell surface changes for other S-layer forming bacteria.

## Materials and methods

### Protein isolation via LiCl

Surface proteins were isolated from biological triplicates using a modified LiCl S-layer extraction protocol (Johnson et al., 2013) adapted for downstream quantitative proteomics. *L. acidophilus* NCFM (NCK56) was grown statically in 800 ml of de Man, Rogosa, and Sharpe broth (MRS, Difco) at 37°C. Cultures were sampled at logarithmic (log, 6 h, 500 ml) and early stationary phase (stat, 12 h, 300 ml) then processed immediately. All subsequent centrifugation steps were performed at 4°C. Briefly, bacterial cells were centrifuged at 3,220 × *g* for 10 min, then washed twice with cold PBS pH 7.4 (Gibco, 4°C). Pellets were resuspended in 5M LiCl (4°C) for 15 min with repeated agitation, then centrifuged at 7,441 × *g* for 10 min. Supernatants were transferred to Spectra/Por membrane tubing (6–8 kD, Spectrum Laboratories, Inc.) and dialyzed against cold distilled water (4°C) for 24 h with gentle stirring and frequent water changes. Overnight protein precipitates were centrifuged at 22,789 × *g* for 30 min, then resuspended in 1M LiCl (4°C) for 15 min with repeated agitation. Suspensions were centrifuged at 22,789 × *g* for 30 min to separate major Slps from proteins associated with the S-layer. Subsequent supernatants containing the S-layer associated proteins were transferred to Spectra/Por membrane tubing (6–8 kD) and again dialyzed against cold distilled water (4°C) for 24 h with gentle stirring and frequent water changes. Precipitates were harvested via centrifugation at 22,789 × *g* for 30 min, then concentrated in 1 ml distilled water. Final suspensions were pelleted in 1.5 ml microcentrifuge tubes at 16,873 × *g* for 30 min, then stored at −80°C or visualized via SDS-PAGE using precast 4–20% Precise Tris-Hepes protein gels (Thermo Scientific) stained with AcquaStain (Bulldog Bio). Frozen protein pellets were submitted to the Genome Center Proteomics Core at the University of California, Davis for proteomic analysis.

### Protein digestion

Protein pellets were solubilized in 100 μL of 6M urea in 50 mM TEAB (triethylammonium bicarbonate) then quantified via Pierce BCA Protein Assay Kit (Thermo Scientific). Digestion was performed on 150 μg of protein. Briefly 200 mM of dithiothreitol (DTT) was added to a final concentration of 5 mM, then incubated for 30 min at 37°C. Next, 20 mM iodoacetamide (IAA) was added to a final concentration of 15 mM, then incubated for 30 min at room temperature. Unreacted IAA was quenched by the addition of 20 μL DTT. Trypsin/Lys-C (Promega) was then added to the sample and incubated for 4 h at 37°C. Samples were diluted to <1M urea by the addition of 50 mM ammonium bicarbonate (AMBIC) and digested overnight at 37°C. The following day, samples were desalted using MacroSpin Column (Nest Group).

### TMT labeling

Desalted peptides were reconstituted in 40 μl of 50 mM TEAB and quantified using Pierce Fluorometric Peptide Assay (Thermo Scientific). Each sample was diluted with 50 mM TEAB to 0.5 μg/μl for a total of 50 μg of peptide per replicate and labeled with TMT 6 Plex Mass Tag Labeling Kit (Thermo Scientific). Briefly, 41 μl of each TMT label (126–131) was added to each digested peptide sample and incubated for 1 h. The reaction was quenched with 8 μl of 5% hydroxylamine and incubated for 15 min. All labeled samples were then mixed together and lyophilized to almost dryness. TMT labeled samples were reconstituted in 0.1% trifluoroacetic acid (TFA) and the pH was adjusted to 2 with 10% TFA. The combined sample (20 μg) was separated into 8 fractions by Pierce High pH Reverse-Phase Peptide Fractionation Kit (Thermo Scientific) with an extra wash before separation to remove excess labels. The 8 fractions were dried almost to completion.

### LC-MS/MS

LC separation was done on a Dionex Nano Ultimate 3000 (Thermo Scientific) with a Thermo EASY-Spray source. Digested peptides were reconstituted in 2% acetonitrile/0.1% TFA and 5 μl of each sample was loaded onto a PepMap 100 Å 3U 75 μm × 20 mm reverse phase trap where they were desalted online before being separated on a 100 Å 2U 50 μm × 150 mm PepMap EASY-Spray reverse phase column. Peptides were eluted using a 70 min gradient of 0.1% formic acid (A) and 80% acetonitrile (B) with a flow rate of 200 nL/min. The separation gradient was run with 2 to 5% B over 1 min, 5 to 10% B over 9 min, 10 to 20% B over for 27 min, 20 to 35% B over 10 min, 35 to 99% B over 10 min, a 2 min hold at 99% B, and finally 99 to 2% B held at 2% B for 5 min.

### MS3 synchronous precursor selection workflow

Mass spectra were collected on a Fusion Lumos Mass Spectrometer (Thermo Fisher Scientific) in a data-dependent MS3 synchronous precursor selection (SPS) method. MS1 spectra were acquired in the Orbitrap, 120 K resolution, 50 ms max inject time, 5 × 10^5^ automatic gain control (AGC). MS2 spectra were acquired in the linear ion trap with a 0.7 Da isolation window, collisionally induced dissociation (CID) fragmentation energy of 35%, turbo scan speed, 50 ms max inject time, 1 × 10^4^ AGC and maximum parallelizable time turned on. MS2 ions were isolated in the iontrap and fragmented with a higher-energy collisional dissociation (HCD) of 65%. MS3 spectra were acquired in the orbitrap with a resolution of 50 K, a scan range of 100–500 Da, 105 ms max inject time and 1 × 10^5^ AGC.

### Protein database searches

Tandem mass spectra were extracted by Proteome Discoverer version 2.1. Charge state deconvolution and deisotoping were not performed. All MS/MS samples were analyzed using Sequest (XCorr Only; Thermo Scientific; version 2.1.0.81). Sequest (XCorr Only) was set up to search all *L. acidophilus* sequences from Uniprot and 110 common laboratory contaminants (http://www.thegpm.org/crap/) plus an equal number of reverse decoy sequences (3,964 total entries) assuming the digestion enzyme trypsin. Sequest (XCorr Only) was searched with a fragment ion mass tolerance of 0.60 Da and a parent ion tolerance of 10.0 PPM. Carbamidomethyl of cysteine and TMT 6 plex of lysine were specified in Sequest (XCorr Only) as fixed modifications. Deamination of asparagine, oxidation of methionine and acetylation of the N-terminus were specified in Sequest (XCorr Only) as variable modifications.

### Quantitative data analysis

Scaffold Q+ (version Scaffold_4.7.5, Proteome Software Inc.) was used to quantitate Label Based Quantitation (iTRAQ, TMT, SILAC, etc.) peptide and protein identifications. Peptide identifications were accepted if they could be established at >95.0% probability by the Scaffold Local FDR algorithm. Protein identifications were accepted if they could be established at >99.0% probability and contained at least 2 identified peptides. Protein probabilities were assigned by the Protein Prophet algorithm (Nesvizhskii et al., 2003). Proteins that contained similar peptides and could not be differentiated based on MS/MS analysis alone were grouped to satisfy the principles of parsimony. Proteins sharing significant peptide evidence were grouped into clusters. Data was extracted from ScaffoldQ+ using the Raw Data Report export and filtered for contaminants and residual Slps. Resulting file was further analyzed with the SafeQuant R package v.2.3.1 (https://github.com/eahrne/SafeQuant/) using the following command line parameters “–AR –EX 1,2,3:4,5,6” (Glatter et al., 2012; Ahrné et al., 2013, 2016).

### Functional classification analysis

Functional categories were assigned using DAVID Bioinformatics Resources 6.8 Functional Annotation Tool (Huang da et al., 2009a,b). Enrichment analyses were performed by searching differentially expressed proteins (*q*-Value < 0.05) against a background of all isolated/identified proteins using default parameters. Cytoplasmic proteins and proteins possessing signal peptides were selected as the focus due to their frequency and absence of overlap with each other.

## Results

### Visualization and quantification of proteins associated with the *L. acidophilus* S-layer

**Figure 1:**
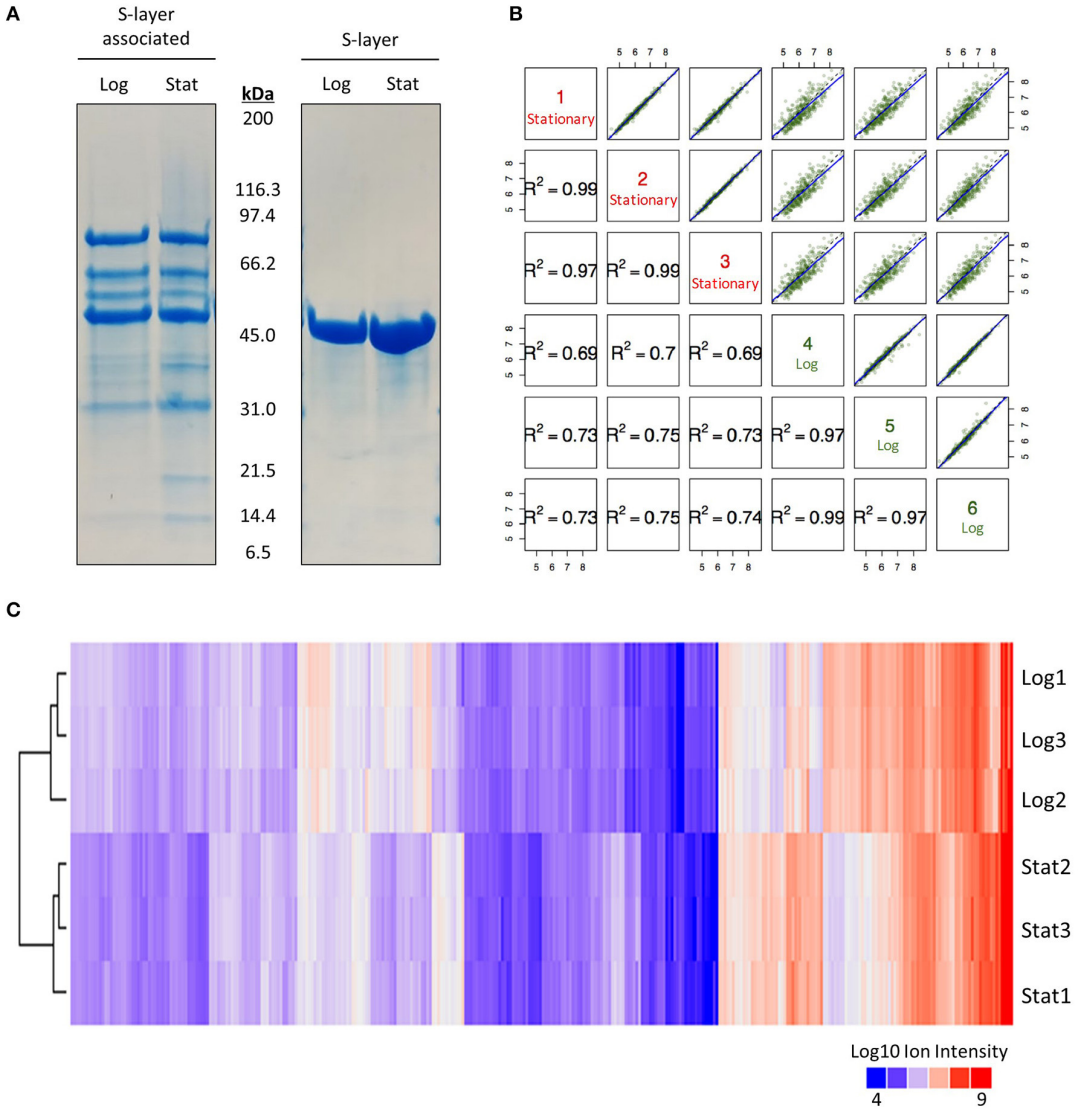
SDS-PAGE and quantitative proteomic results**. (A)** SDS-PAGE gel used to visualize the effect of growth phase on the S-layer and S-layer associated proteome of *Lactobacillus acidophilus* NCFM at 6 h (log) and 12 h (stat). **(B)** Correlation plot of multiplexing mass spectrometry data for all identified S-layer associated proteins in logarithmic and early stationary growth phases. **(C)** Clustering of Log10 ion intensities for all identified proteins in logarithmic and early stationary growth phases.

A previously described LiCl extraction protocol was used to enrich for proteins non-covalently associated with the cell surface of *L. acidophilus* NCFM (Johnson et al., 2013). Log and early stationary S-layer and S-layer associated proteins were initially visualized via SDS-PAGE gel (Figure 1A). Banding patterns and abundances of the S-layer associated protein fraction appeared dissimilar enough to merit quantitative proteomic analysis. We chose to use a TMT 6 Plex in conjunction with a synchronous precursor selection (SPS)-based MS3 technology to eliminate the common interference effects associated with MS2 (Ting et al., 2011). Multiplexed reactions produced very tight biological replicates (*R*^2^ ≥ 0.97) with clear growth-stage-dependent effects (Figures 1B,C). Through this approach, we identified 352 proteins of which 276 were differentially expressed (Figure 1C and Supplementary Table 1). Proteins with a *q*-Value < 0.05 were considered significant.

### Distribution of S-layer associated proteins based on functional categories

**Figure 2:**
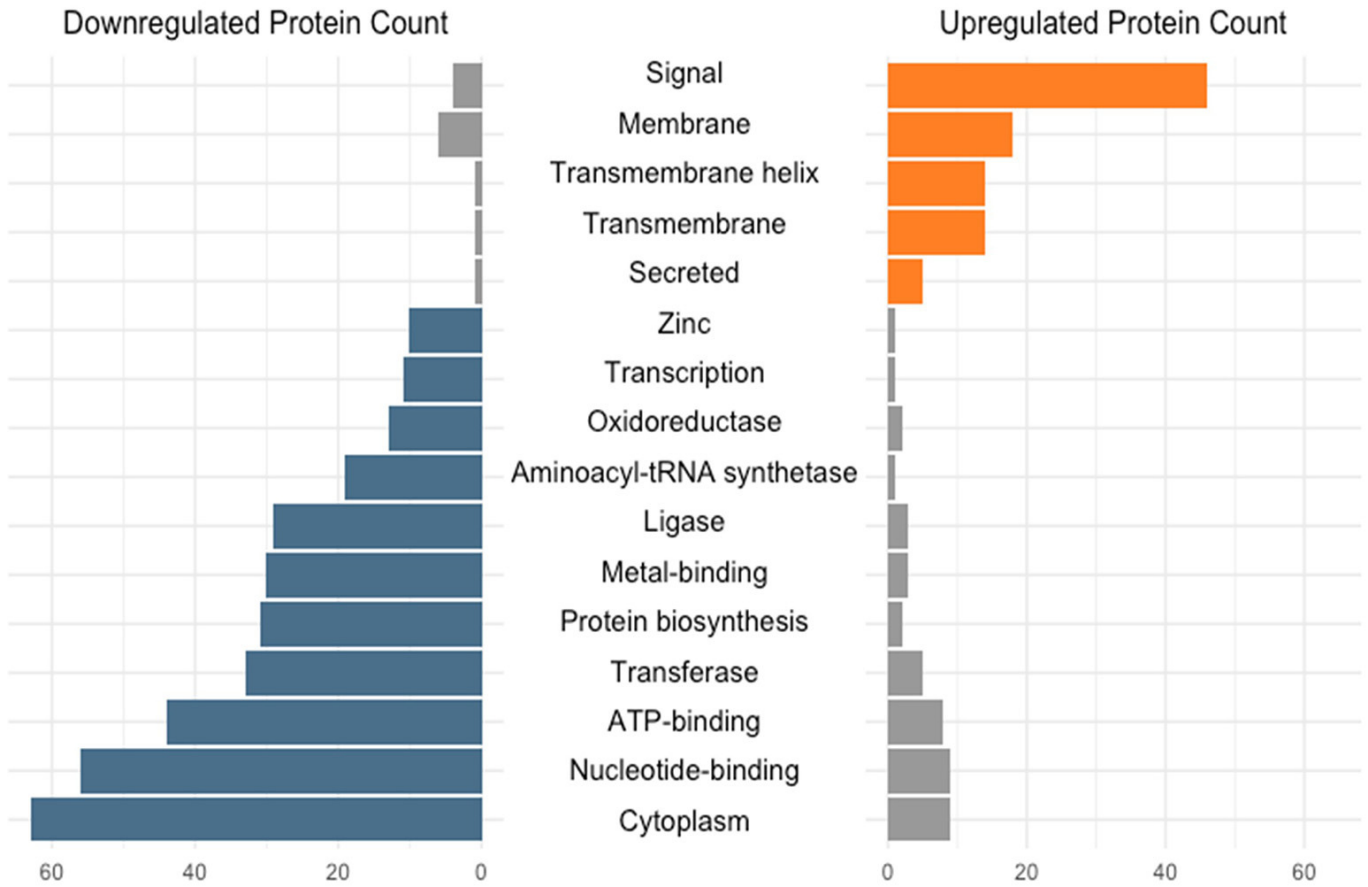
Number of differentially expressed (*q* < 0.05) early stationary phase S-layer associated proteins grouped based on functional category. Categories were assigned via the DAVID algorithm using default parameters.

**Figure 3:**
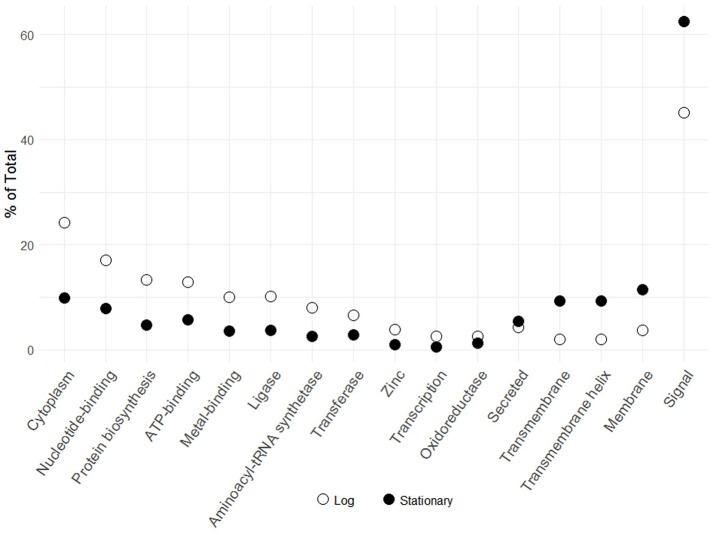
Percent abundance of logarithmic and early stationary phase S-layer associated protein functional categories identified in Figure 2. Functional categories were assigned via the DAVID algorithm using default parameters.

The transition from log to stationary phase produced notable fluctuations in protein counts between diverse functional categories (Figure 2). Of the 276 afflicted proteins, 50 were shown to possess signal peptides, 46 of which were significantly upregulated. Signal sequences are indicative of proteins destined to either be secreted or incorporated into cell wall or cell membrane components (Kleerebezem et al., 2010). Additional upregulated functional categories include 18 membrane, 14 transmembrane helix, 14 transmembrane, and five secreted proteins. The most frequently downregulated functional categories included 63 cytoplasmic proteins, followed by nucleotide-binding and ATP-binding with 56 and 44 proteins, respectively. The greater number of proteins within these categories did not necessarily correlate with overall abundance. Figure 3 illustrates the percent abundance of each functional category in comparison to the total. Unsurprisingly, proteins possessing signal peptides were dominant in both log (45.1%) and stationary (62.4%) growth phases. Although 24.2% of log phase proteins were classified as cytoplasmic, the category is mainly composed of a few highly abundant moonlighting proteins detailed in Table 1. These proteins are predicted to be cytoplasmic, but have been shown to possess secondary function on the bacterial cell surface (Bendtsen et al., 2005; Wang et al., 2014). Many of the remaining cytoplasmic proteins were in relatively low abundance and possibly a result of low level cell lysis.

**Table 1:** The 25 most abundant S-layer associated proteins in logarithmic phase and early stationary phase.

**Gene**	**Predicted function**	**Log2 (Stat/Log)**	***q*-Value**	**Functional category**
**LOGARITHMIC**				
LBA1578	Putative serine protease	**−0.52**	**0.077187183**	Signal
LBA0695	Putative uncharacterized protein	**0.19**	**0.719561982**	Signal
LBA1426	Putative uncharacterized protein	**1.86**	**8.30E-06**	Signal
^*^LBA0889	Eno—Enolase	**−1.47**	**8.97E-06**	Cytoplasm
^*^LBA0846	Tig—Trigger factor	**−1.03**	**0.000306568**	Cytoplasm
LBA1567	Aminopeptidase	**1.07**	**0.00041098**	Signal
LBA1162	Asparagine–tRNA ligase	**−2.33**	**2.76E-06**	Cytoplasm
LBA0223	CdpA—Cell separation protein	**−0.31**	**0.505522785**	Other
LBA1512	PrtP	**0.45**	**0.020404475**	Other
^*^LBA1599	FbaA—Fructose-bisphosphate aldolase	**−1.70**	**5.18E-06**	Other
^*^LBA0289	FusA—Elongation factor G	**−0.88**	**0.002079319**	Cytoplasm
LBA1611	FmtB—Surface protein	2.59	0.027027964	Other
LBA0858	Penicillin-binding protein	0.66	0.003522194	Signal
^*^LBA0698	Glyceraldehyde-3-p dehydrogenase	−0.64	0.003045344	Other
LBA0957	KpyK—Pyruvate kinase	−0.27	0.204266652	Other
^*^LBA0845	Tuf—Elongation factor Tu	−1.36	4.72E-05	Cytoplasm
LBA1918	LysA—Lysin	−1.75	1.49E-05	Signal
LBA1763	PepF—Oligopeptidase	−2.65	8.61E-06	Other
LBA0185	GpmA−2, 3-bisphosphoglycerate-dependent phosphoglycerate mutase	−1.80	8.02E-06	Other
LBA0222	Putative uncharacterized protein	1.19	0.003670271	Signal
LBA0831	BipA—GTP-binding protein-BipA-EF-TU family	−2.30	5.14E-06	Other
LBA1270	RpsB−30S ribosomal protein S2	−0.54	0.005909604	Other
LBA1225	Putative uncharacterized protein	−0.15	0.646538001	Signal
LBA1262	ProS—Proline–tRNA ligase	−0.83	0.000793245	Cytoplasm
LBA1543	ThrS—Threonine–tRNA ligase	−1.61	2.28E-05	Cytoplasm
**EARLY STATIONARY**				
LBA1426	Putative uncharacterized protein	1.86	8.30E-06	Signal
LBA1578	Putative serine protease	−0.52	0.077187183	Signal
LBA1611	FmtB -Surface protein	2.59	0.027027964	Other
LBA0695	Putative uncharacterized protein	0.19	0.719561982	Signal
LBA1567	Aminopeptidase	1.07	0.00041098	Signal
LBA1539	Putative uncharacterized protein	3.96	2.53E-06	Signal
LBA1690	Putative membrane protein	2.04	1.19E-05	Signal
LBA1512	PrtP	0.45	0.020404475	Other
LBA0222	Putative uncharacterized protein	1.19	0.003670271	Signal
LBA0858	Penicillin-binding protein	0.66	0.003522194	Signal
LBA1612	Fibrinogen-binding protein	2.84	0.015555274	Signal
LBA1020	Putative mucus binding protein	3.27	0.000326332	Signal
LBA0292	RplD−50S ribosomal protein L4	2.88	3.16E-06	Other
LBA0223	CdpA—Cell separation protein	−0.31	0.505522785	Other
^*^LBA0846	Tig—Trigger factor	−1.03	0.000306568	Cytoplasm
LBA0370	RplL−50S ribosomal protein L7/L12	1.41	0.002624707	Other
^*^LBA0889	Eno—Enolase	−1.47	8.97E-06	Cytoplasm
LBA0864	Putative uncharacterized protein	0.51	0.023311864	Signal
LBA0191	Putative fibronectin domain	2.00	1.28E-05	Signal
LBA1739	Putative uncharacterized protein	0.99	0.000538559	Signal
LBA1300	OppA—Oligopeptide ABC trasporter substrate binding protein	1.93	5.63E-06	Signal
LBA0957	KpyK—Pyruvate kinase	−0.27	0.204266652	Other
LBA0778	AtpD—ATP synthase subunit beta	0.61	0.001313383	Other
^*^LBA0289	FusA—Elongation factor G	−0.88	0.002079319	Cytoplasm
^*^LBA0698	Glyceraldehyde-3-p dehydrogenase	**−0.64**	**0.003045344**	Other

**Figure 4:**
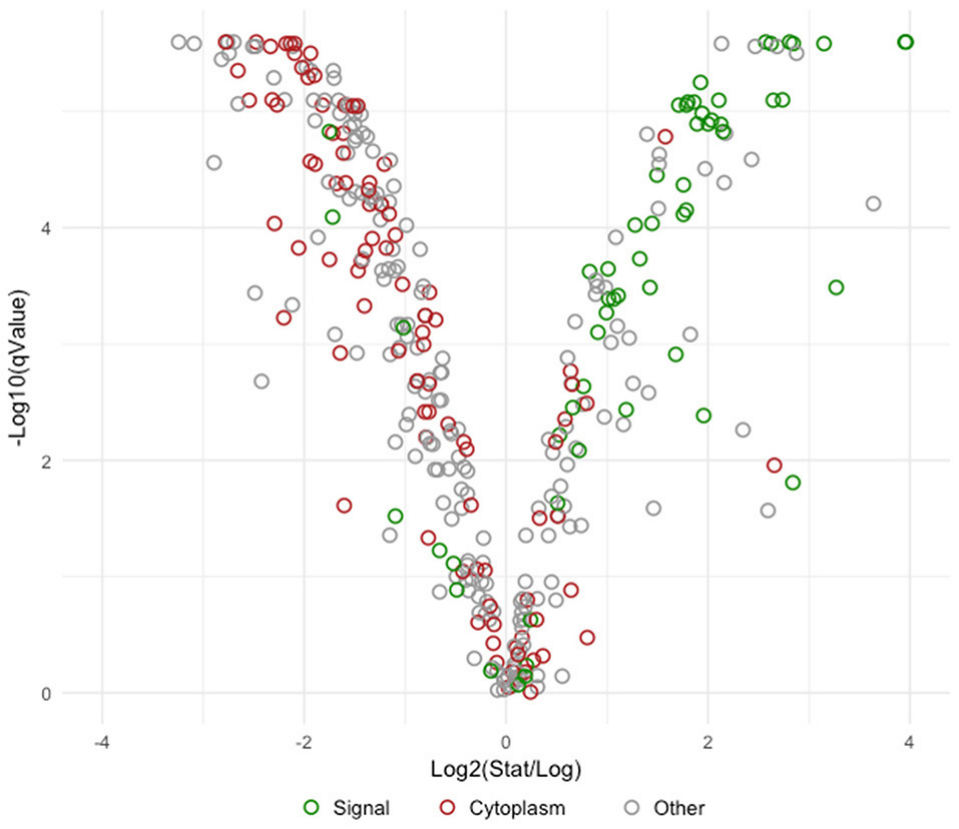
Volcano plot comparing Log2 fold change to -Log10 statistical significance. Visually depicts the overall change in the early stationary phase S-layer associated proteome in comparison to logarithmic phase. Proteins possessing signal peptides are colored in green, those predicted to be cytoplasmic are colored red, and remaining proteins are colored gray.

* *Previously reported moonlighting function. Red and green coloring corresponds with volcano plot in Figure 4*.

Overall differences were visualized by plotting Log2 fold change against the statistical significance (–Log10 *q*-Values) for all identified proteins (Figure 4). Proteins possessing signal peptides were colored in green while those predicted to be cytoplasmic were colored red. As cells switched from log to stationary phase, there was a clear trend in the upregulation of signal proteins and the downregulation of cytoplasmic proteins. The most significantly affected proteins (|Log2 fold change| > 2 and a *q*-Value < 1E-10) are presented in Table 2. Noteworthy, a putative uncharacterized protein (LBA1539), a 50S ribosomal protein (LBA0292), a putative membrane protein (LBA1690), and a putative fibronectin domain (LBA0191) were substantially upregulated, leading them to become amongst the most abundant proteins on the stationary cell surface (Table 1). Alternatively, an asparagine—tRNA ligase (LBA1162), oligopeptidase PepF (LBA1763), and GTP-binding protein BipA (LBA0831) were initially amongst the most abundant log phase proteins (Table 1) but were vastly downregulated during the transition to stationary phase.

**Table 2:** S-layer associated proteins most significantly affected by growth phase (|Log2 fold change| > 2 and a *q*-Value < 1E−10).

**Gene**	**Predicted function**	**Log2 (Stat/Log)**	***q*-Value**	**Functional category**
**UPREGULATED**				
LBA1539	Putative uncharacterized protein	3.96	2.53E-06	Signal
LBA1744	Putative glycosidase	3.95	2.53E-06	Signal
LBA1219	Putative lipase	3.64	6.21E-05	Other
LBA0112	Putative glutamine ABC transporter	3.15	2.61E-06	Signal
LBA0292	RplD−50S ribosomal protein	2.88	3.16E-06	Other
LBA0361	ABC transporter	2.84	2.61E-06	Signal
LBA0014	Putative alkylphosphonate ABC transporter	2.81	2.53E-06	Signal
LBA1601	Putative cell surface protein	2.74	7.95E-06	Signal
LBA1011	Putative uncharacterized protein	2.68	2.76E-06	Other
LBA0136	Putative uncharacterized protein	2.65	8.02E-06	Signal
LBA1603	VanY—D-alanyl-d-alanine carboxypeptidase	2.62	2.61E-06	Signal
LBA0134	GlnP—Glutamine ABC transporter permease protein	2.57	2.53E-06	Signal
LBA1509	Penicillin-binding protein	2.47	2.76E-06	Other
LBA0040	Putative uncharacterized protein	2.43	2.59E-05	Other
LBA1010	Secreted protein	2.17	1.54E-05	Other
LBA0083	HtrA—Putative heat shock related serine protease	2.16	4.10E-05	Other
LBA0046	Putative uncharacterized protein	2.15	1.49E-05	Signal
LBA0360	RplA−50S ribosomal protein	2.13	2.61E-06	Other
LBA1850	LysM—Putative aggregation promoting protein	2.13	1.29E-05	Signal
LBA1654	PspC—Putative surface protein	2.11	8.02E-06	Signal
LBA1690	Putative membrane protein	2.04	1.19E-05	Signal
LBA0191	Putative fibronectin domain	2.00	1.28E-05	Signal
**DOWNREGULATED**				
LBA0936	AspS—Aspartate–tRNA ligase	−2.02	4.2E-06	Cytoplasm
LBA1259	NusA—Transcription termination/antitermination protein	−2.09	2.61E-06	Cytoplasm
LBA0273	TrcF—Transcription-repair-coupling factor	−2.1	3.16E-06	Cytoplasm
LBA0997	Aluminum resistance protein	−2.11	2.76E-06	Other
LBA0390	TsaD—tRNA N6-adenosine threonylcarbamoyltransferase	−2.14	2.61E-06	Cytoplasm
LBA0794	ValS—Valine–tRNA ligase	−2.18	2.61E-06	Cytoplasm
LBA0159	Putative uncharacterized protein	−2.19	7.94E-06	Other
LBA1617	LeuS—Leucine–tRNA ligase	−2.27	8.78E-06	Cytoplasm
LBA0366	PhoU—Phosphate-specific transport system accessory protein	−2.29	9.21E-05	Cytoplasm
LBA0831	BipA—GTP-binding protein-BipA-EF-TU family	−2.3	5.14E-06	Other
LBA0417	AlaS—Alanine–tRNA ligase	−2.32	7.95E-06	Cytoplasm
LBA1162	Asparagine–tRNA ligase	−2.33	2.76E-06	Cytoplasm
^*^LBA0285	RpoC—DNA-directed RNA polymerase subunit beta'	−2.48	2.76E-06	Other
LBA0657	Putative tRNA (cytidine(34)-2′-O)-methyltransferase	−2.48	2.53E-06	Cytoplasm
^*^LBA0284	RpoB—DNA-directed RNA polymerase subunit beta	−2.51	2.76E-06	Other
LBA1248	GrpE—Protein GrpE	−2.54	8.02E-06	Cytoplasm
LBA1763	PepF—Oligopeptidase	−2.65	8.61E-06	Other
LBA0261	GlyA—Serine hydroxymethyltransferase	−2.66	4.46E-06	Cytoplasm
LBA0131	Ribose-p pyrokinase	−2.7	2.53E-06	Other
LBA0908	Fumarate reductase flavoprotein	−2.74	3.16E-06	Other
LBA0233	PyrG—CTP synthase	−2.76	2.53E-06	Other
LBA0817	IleS—Isoleucine–tRNA ligase	−2.78	2.53E-06	Cytoplasm
LBA1562	Fhs2—Formate–tetrahydrofolate ligase 2	−2.82	3.57E-06	Other
LBA1321	Fmt—Methionyl-tRNA formyltransferase	−2.89	2.76E-05	Other
LBA1891	PurB—Adenylosuccinate lyase	−3.09	2.61E-06	Other
LBA0132	TetR—Putative transcriptional regulator	−3.24	2.53E-06	Other

* *Previously reported moonlighting function. Red and green coloring corresponds with volcano plot in Figure 4*.

### Cluster analysis of the most abundant proteins in logarithmic and early stationary growth phases

**Figure 5:**
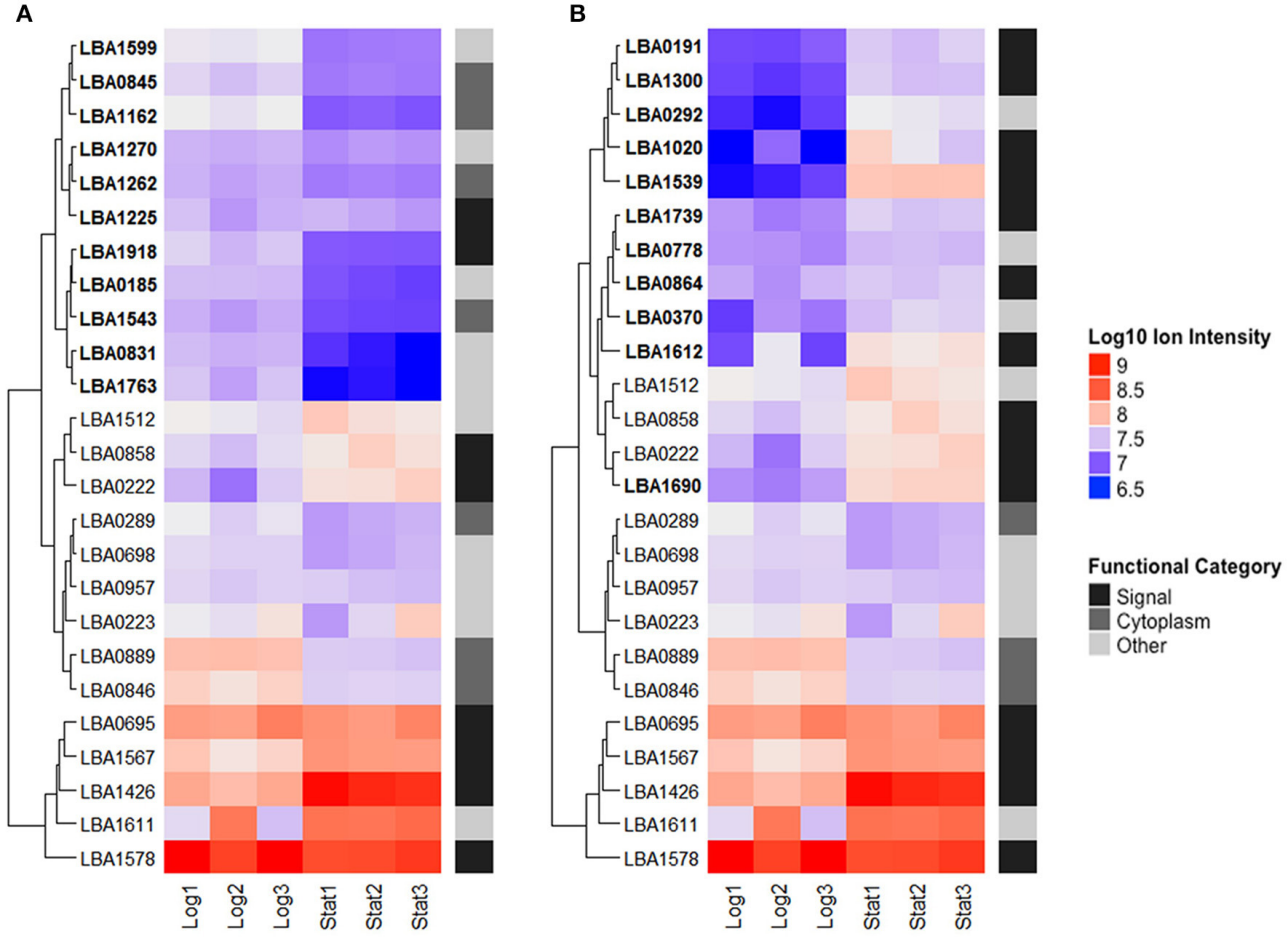
The top 25 most abundant S-layer associated proteins in logarithmic **(A)** and early stationary **(B)** growth phases. Proteins are clustered based on their Log10 ion intensities for both growth phases. Functional categories are labeled on the right. Unique proteins are bolded. Additional information about these proteins can be found in Table 1.

The 25 most abundant log and stationary phase proteins (Table 1 and Figure 5), though only accounting for 7% of the total number of identified proteins, encompassed 66 and 74% of the total abundance. A clustering of these proteins using their Log10 ion intensities is depicted in Figure 5. Unique proteins are shown in bold. Proteins are once again classified as either signal, cytoplasmic or other. Additional details about these proteins can be found in Table 1. Despite considerable differences, a conserved bottom branch comprised of five proteins (LBA1578, LBA1611, LBA1426, LBA1567, and LBA0695) with consistent high expression appears in both growth phases. Of these five proteins, only surface protein FmtB (LBA1611) does not possess a signal peptide. Three of these proteins, putative uncharacterized protein LBA1426, surface protein FmtB (LBA1611), and an aminopeptidase (LBA1567), although all prominent populations in log phase, were significantly upregulated in stationary phase. In fact, LBA1578, a putative serine protease, was replaced by uncharacterized LBA1426 as the overall most abundant protein when cells transitioned into stationary phase. Though unaffected by growth phase, putative uncharacterized protein LBA0695, cell separation protein CdpA (LBA0223), PrtP (LBA1512), and pryruvate kinase (LBA0957) remained dominant in both growth phases (Figure 5). Trigger factor (LBA0846), enolase (LBA0889), and elongation factor G (LBA0289), all well-established moonlighting proteins (Bendtsen et al., 2005; Wang et al., 2014), were prevalent in both growth phases despite being significantly downregulated in stationary phase (Figure 5). Alternatively, cytoplasmic proteins dominant only in log phase included asparagine, proline, and threonine—tRNA ligases (LBA1162, LBA1262, and LBA1543) in addition to elongation factor Tu (LBA0846), another recognized moonlighting protein. A direct comparison of log (Figure 5A) and stationary (Figure 5B) growth phases showed the number of abundant proteins possessing signal peptides nearly doubled in stationary phase. Twelve of these proteins were significantly upregulated (*q*-Value < 0.05), of which four increased >2-Log2 fold (LBA1539, LBA1020, LBA1612, and LBA1690). Despite being dominant populations on the cell surface, many of these proteins remain uncharacterized (LBA1225, LBA0222, LBA0695, LBA1426, LBA1739, LBA0864, and LBA1539).

## Discussion

Molecular-based approaches have become increasingly applied to probiotic research in an effort to define the underlying mechanisms of probiotic activity (Marco et al., 2006). Several recent studies have targeted proteins associated with the *L. acidophilus* NCFM S-layer due to their exterior location and potential to mediate probiotic-host interactions (Johnson et al., 2013, 2017; Hymes et al., 2016; Johnson and Klaenhammer, 2016). Although, previous research has investigated the influence of growth phase on the lactobacilli proteome (Kelly et al., 2005; Cohen et al., 2006), none obtained the depth or quantitative accuracy we generated via the elimination of a 2-DE gel step and use of isobaric labeling combined with MS3 identification. Through this approach we demonstrated that the *L. acidophilus* cell surface is far more diverse and complex than previously described.

We strategically chose to examine log and early stationary growth phases to limit potential cell death/lysis, and thus intracellular protein contamination. Nevertheless, cytoplasmic proteins were still the most prevalent functional category. These results are consistent with several other studies which routinely found cytoplasmic proteins assuming secondary functions on probiotic cell surfaces (Beck et al., 2009; Johnson et al., 2013; Espino et al., 2015; Celebioglu and Svensson, 2017; Celebioglu et al., 2017). Moonlighting proteins tend to be associated with pathogenic functions such as adhesion to host epithelia and extracellular matrices, along with modulation of the immune response. However, moonlighting proteins in probiotics also share many of these same characteristics (Wang et al., 2014). In a recent study using trypsin shaving of probiotic *Lactobacillus rhamnosus* GG and dairy strain *L. rhamnosus* Lc705, 77 and 88% of surface-exposed proteins were revealed to be cytoplasmic, respectively. Interestingly, the presence of many of these putative moonlighting proteins was predicted to be dependent on growth stage or pH (Espino et al., 2015). Within our own data, a number of well-known, highly abundant moonlighting proteins including enolase, trigger factor, fructose-bisphosphate aldolase, elongation factor Tu, elongation factor G, and glyceraldehyde-3-p dehydrogenase, were downregulated in stationary phase. This contrasted previous studies with *Bacillus subtilis* and *Lactobacillus salivarius*, which demonstrated increased non-classical protein secretion during stationary phase (Bendtsen et al., 2005; Kelly et al., 2005). Since the secondary function of most characterized cell surface moonlighting proteins is adherence, one might predict decreased adhesive capacity of stationary phase cells (Amblee and Jeffery, 2015). Many of the remaining cytoplasmic proteins were present in low relative abundance and predicted to be a result of minor cell lysis. Nonetheless, their detection emphasizes the power and robustness of the MS3 technology. In fact, MS3 yielded almost a tenfold increase in protein identification in comparison to previous *L. acidophilus* exoproteome studies (Johnson et al., 2013; Celebioglu and Svensson, 2017).

Alternatively, proteins that were upregulated to exceptionally high abundance in stationary phase include a putative membrane protein (LBA1690), a putative fibronectin domain protein (LBA0191), a 50S ribosomal protein (LBA0292), and a previously uncharacterized protein (LBA1539). The membrane protein and fibronectin domain protein have both been shown to play a role in adhesion (Azcarate-Peril et al., 2009; Hymes et al., 2016). LBA1690 was insertionally inactivated, resulting in a 30 and 68% reduction in Caco-2 and mucin adhesion, respectively (Azcarate-Peril et al., 2009). LBA0191 was deleted from the *L. acidophilus* chromosome resulting in a 47 and 72% reduction in mucin and fibronectin adhesion, respectively (Hymes et al., 2016). A putative mucus binding protein (LBA1020) and fibrinogen-binding protein (LBA1612), though not quite as impactful, were also considerably upregulated in stationary phase. It is possible that these proteins may offset the potential binding-loss stemming from downregulated moonlighting proteins.

The 50S ribosomal protein (LBA0292) does not have a characterized cell surface function but has been located on the exterior of *Enterococcus faecalis* (Bøhle et al., 2011) and *Staphylococcus aureus* (Dreisbach et al., 2010) and shown to play many roles beyond translation (Warner and Mcintosh, 2009). Uncharacterized protein LBA1539 was the most upregulated protein in our dataset as well as one of the most abundant. In fact, amongst the top 25 most abundant log and stationary phase proteins, there are an additional six putative uncharacterized proteins (LBA1225, LBA0222, LBA0695, LBA1426, LBA1739, and LBA0864). These proteins are in high abundance on the cell surface, and likely make direct physical contact with the host gastrointestinal tract, yet we have no knowledge of their functional role. BLASTP and PFAM searches revealed that many of these proteins are highly conserved amongst the *Lactobacillus* genus and possess several interesting domains including: SH3-like (PF13457), bacterial Ig-like (PF07523), SLAP (PF03217), and CAP (PF00188). SH3 domains function predominately in cell wall turnover (Kleerebezem et al., 2010). The SLAP domain, though distantly related to the SH3 domain, is responsible for the extracellular Slp scaffold and non-covalent attachment of secreted proteins (Boot et al., 1995; Johnson et al., 2015).

LBA0695 and LBA1426 cluster with a set of proteins that have consistently high expression in both log and stationary growth phases. Within the literature there is little mention of these two proteins outside of the group 3 bacterial Ig-like domain on LBA0695 (Johnson et al., 2015) and the upregulation of LBA1426 when exposed to bile (Pfeiler et al., 2007). Although LBA1426 was highly expressed during log phase, it underwent significant upregulation in stationary phase, eventually replacing a putative serine protease (LBA1578) as the most abundant protein within our dataset. However, the serine protease was constitutively expressed throughout both growth phases. This protein was recently characterized in *L. acidophilus* NCFM and shown to have distinct effects on cellular morphology leading to altered binding ability, immunomodulatory properties, and a hypothesized role in protein turnover and display on the cell surface (Johnson et al., 2017). Because these three proteins are consistently highly expressed and known to localize to the cell surface, they may prove to be interesting targets for engineering, specifically for the display of recombinant proteins for vaccination. In general, LABs are promising antigen delivery candidates for processing and presentation by the immune system (Wells and Mercenier, 2008). *L. acidophilus* NCFM is of particular interest due to its ability to survive gastric passage and potential to increase the response to oral antigens (Sanders and Klaenhammer, 2001). In past studies, S-layer protein A (SlpA) and enolase have been exploited for this purpose (Douglas and Klaenhammer, 2011; Kajikawa et al., 2015; O'Flaherty and Klaenhammer, 2016), though novel proteins may prove to be valuable contenders for future research.

The *L. acidophilus* cell surface is clearly modulated by growth phase, thus so are the proteins presented to the host. In a human trial administering probiotic *Lactobacillus plantarum*, different growth phases yielded vastly diverse mucosal responses (van Baarlen et al., 2009). Stationary phase cells were correlated with host genes regulating immune responses and stimulation of cellular physiology, while log phase cells were associated with nucleic acid metabolism, cytoplasm organization and biogenesis (van Baarlen et al., 2009). These distinctions were hypothesized to be a result of cell envelope and exopolysaccharide-associated functions and highlight the importance of probiotic cell surface research. Within our own study, the transition into stationary phase was associated with the upregulation of extracellular proteins and thus a shift in focus to the cell exterior. Understanding the role of these proteins in probiotic function may assist in illuminating mechanisms responsible for their beneficial effects and further research on the microbe-host crosstalk occurring within the confines of the human gastrointestinal tract.

## Conclusions

Significant alterations to the *L. acidophilus* surface-associated proteome were demonstrated as cells transitioned from log to stationary phase. Both condition-dependent and stably expressed proteins were identified as candidates for functional characterization and cell surface engineering. Additionally, this study establishes a framework for future research of S-layer associated proteins beyond *L. acidophilus*. The combination of multiplexing and MS3 identification yielded reproducible data with noteworthy condition-dependent effects, thus we encourage its use in future studies. Overall, surface protein modulation remains an important factor in probiotic optimization. Furthering this research is imperative for identifying the genotypes and phenotypes conferred by probiotic cell surface proteins to enhance their delivery, persistence, and general efficacy.

## Author contributions

CK, YJG, and RB designed the study; CK carried out the work, analyzed the results, and prepared the manuscript under the supervision of RB, SOF, and YJG.

### Conflict of interest statement

The authors declare that the research was conducted in the absence of any commercial or financial relationships that could be construed as a potential conflict of interest.

## Supplementary material

Supplementary Table 1 Additional information and Uniprot Accession numbers for all proteins identified in this study.

## References

[B1] Ahrné E. Glatter T. Viganò C. Von Schubert C. Nigg E. A. Schmidt A. (2016). Evaluation and improvement of quantification accuracy in isobaric mass tag-based protein quantification experiments. J. Proteome Res. 15, 2537–2547. 10.1021/acs.jproteome.6b00066 27345528

[B2] Ahrné E. Molzahn L. Glatter T. Schmidt A. (2013). Critical assessment of proteome-wide label-free absolute abundance estimation strategies. Proteomics 13, 2567–2578. 10.1002/pmic.201300135 23794183

[B3] Altermann E. Russell W. M. Azcarate-Peril M. A. Barrangou R. Buck B. L. McAuliffe O.. (2005). Complete genome sequence of the probiotic lactic acid bacterium *Lactobacillus acidophilus* NCFM. Proc. Natl. Acad. Sci. U.S.A. 102, 3906–3912. 10.1073/pnas.0409188102 15671160 PMC554803

[B4] Amblee V. Jeffery C. J. (2015). Physical features of intracellular proteins that moonlight on the cell surface. PLoS ONE 10:e0130575. 10.1371/journal.pone.0130575 26110848 PMC4481411

[B5] Azcarate-Peril M. A. Tallon R. Klaenhammer T. R. (2009). Temporal gene expression and probiotic attributes of *Lactobacillus acidophilus* during growth in milk. J. Dairy Sci. 92, 870–886. 10.3168/jds.2008-1457 19233780

[B6] Beck H. C. Madsen S. M. Glenting J. Petersen J. Israelsen H. Nørrelykke M. R.. (2009). Proteomic analysis of cell surface-associated proteins from probiotic *Lactobacillus plantarum*. FEMS Microbiol. Lett. 297, 61–66. 10.1111/j.1574-6968.2009.01662.x 19527296

[B7] Bendtsen J. D. Kiemer L. Fausbøll A. Brunak S. (2005). Non-classical protein secretion in bacteria. BMC Microbiol. 5:58. 10.1186/1471-2180-5-58 16212653 PMC1266369

[B8] Bøhle L. A. Riaz T. Egge-Jacobsen W. Skaugen M. Busk O. L. Eijsink V. G.. (2011). Identification of surface proteins in *Enterococcus faecalis* V583. BMC Genomics 12:135. 10.1186/1471-2164-12-135 21362161 PMC3059304

[B9] Boot H. J. Kolen C. P. Pouwels P. H. (1995). Identification, cloning, and nucleotide sequence of a silent S-layer protein gene of *Lactobacillus acidophilus* ATCC 4356 which has extensive similarity with the S-layer protein gene of this species. J. Bacteriol. 177, 7222–7230. 10.1128/jb.177.24.7222-7230.1995 8522531 PMC177603

[B10] Bron P. A. Van Baarlen P. Kleerebezem M. (2012). Emerging molecular insights into the interaction between probiotics and the host intestinal mucosa. Nat. Rev. Microbiol. 10, 66–78. 10.1038/nrmicro2690 22101918

[B11] Celebioglu H. U. Olesen S. V. Prehn K. Lahtinen S. J. Brix S. Abou Hachem M.. (2017). Mucin- and carbohydrate-stimulated adhesion and subproteome changes of the probiotic bacterium *Lactobacillus acidophilus* NCFM. J. Proteomics 163, 102–110. 10.1016/j.jprot.2017.05.015 28533178

[B12] Celebioglu H. U. Svensson B. (2017). Exo- and surface proteomes of the probiotic bacterium *Lactobacillus acidophilus* NCFM. Proteomics 17:1700019. 10.1002/pmic.201700019 28393464

[B13] Christoforou A. L. Lilley K. S. (2012). Isobaric tagging approaches in quantitative proteomics: the ups and downs. Anal. Bioanal. Chem. 404, 1029–1037. 10.1007/s00216-012-6012-9 22580419

[B14] Cohen D. P. Renes J. Bouwman F. G. Zoetendal E. G. Mariman E. de Vos W. M.. (2006). Proteomic analysis of log to stationary growth phase *Lactobacillus plantarum* cells and a 2-DE database. Proteomics 6, 6485–6493. 10.1002/pmic.200600361 17115453

[B15] Douglas G. L. Klaenhammer T. R. (2011). Directed chromosomal integration and expression of the reporter gene gusA3 in *Lactobacillus acidophilus* NCFM. Appl. Environ. Microbiol. 77, 7365–7371. 10.1128/AEM.06028-11 21873486 PMC3194874

[B16] Dreisbach A. Hempel K. Buist G. Hecker M. Becher D. van Dijl J. M. (2010). Profiling the surfacome of *Staphylococcus aureus*. Proteomics 10, 3082–3096. 10.1002/pmic.201000062 20662103

[B17] Espino E. Koskenniemi K. Mato-Rodriguez L. Nyman T. A. Reunanen J. Koponen J.. (2015). Uncovering surface-exposed antigens of *Lactobacillus rhamnosus* by cell shaving proteomics and two-dimensional immunoblotting. J. Proteome Res. 14, 1010–1024. 10.1021/pr501041a 25531588

[B18] Fagan R. P. Fairweather N. F. (2014). Biogenesis and functions of bacterial S-layers. Nat. Rev. Microbiol. 12, 211–222. 10.1038/nrmicro3213 24509785

[B19] Glatter T. Ludwig C. Ahrné E. Aebersold R. Heck A. J. Schmidt A. (2012). Large-scale quantitative assessment of different in-solution protein digestion protocols reveals superior cleavage efficiency of tandem Lys-C/Trypsin proteolysis over trypsin digestion. J. Proteome Res. 11, 5145–5156. 10.1021/pr300273g 23017020

[B20] Hill C. Guarner F. Reid G. Gibson G. R. Merenstein D. J. Pot B.. (2014). The international scientific association for probiotics and prebiotics consensus statement on the scope and appropriate use of the term probiotic. Nat. Rev. Gastroenterol. Hepatol. 11, 506–514. 10.1038/nrgastro.2014.66 24912386

[B21] Huang da W. Sherman B. T. Lempicki R. A. (2009a). Bioinformatics enrichment tools: paths toward the comprehensive functional analysis of large gene lists. Nucleic Acids Res. 37, 1–13. 10.1093/nar/gkn923 19033363 PMC2615629

[B22] Huang da W. Sherman B. T. Lempicki R. A. (2009b). Systematic and integrative analysis of large gene lists using DAVID bioinformatics resources. Nat. Protoc. 4, 44–57. 10.1038/nprot.2008.211 19131956

[B23] Hymes J. P. Johnson B. R. Barrangou R. Klaenhammer T. R. (2016). Functional analysis of an s-layer-associated fibronectin-binding protein in *Lactobacillus acidophilus* NCFM. Appl. Environ. Microbiol. 82, 2676–2685. 10.1128/AEM.00024-16 26921419 PMC4836419

[B24] Hynönen U. Palva A. (2013). *Lactobacillus* surface layer proteins: structure, function and applications. Appl. Microbiol. Biotechnol. 97, 5225–5243. 10.1007/s00253-013-4962-2 23677442 PMC3666127

[B25] Johnson B. R. Hymes J. Sanozky-Dawes R. Henriksen E. D. Barrangou R. Klaenhammer T. R. (2015). Conserved S-Layer-associated proteins revealed by exoproteomic survey of S-layer-forming lactobacilli. Appl. Environ. Microbiol. 82, 134–145. 10.1128/AEM.01968-15 26475115 PMC4702614

[B26] Johnson B. R. Klaenhammer T. R. (2016). AcmB Is an S-Layer-Associated beta-N-Acetylglucosaminidase and functional autolysin in *Lactobacillus acidophilus* NCFM. Appl. Environ. Microbiol. 82, 5687–5697. 10.1128/AEM.02025-16 27422832 PMC5007774

[B27] Johnson B. R. O'Flaherty S. Goh Y. J. Carroll I. Barrangou R. Klaenhammer T. R. (2017). The S-layer associated serine protease homolog prtx impacts cell surface-mediated microbe-host interactions of *Lactobacillus acidophilus* NCFM. Front. Microbiol. 8:1185. 10.3389/fmicb.2017.01185 28713337 PMC5491966

[B28] Johnson B. Selle K. O'Flaherty S. Goh Y. J. Klaenhammer T. (2013). Identification of extracellular surface-layer associated proteins in *Lactobacillus acidophilus* NCFM. Microbiology 159, 2269–2282. 10.1099/mic.0.070755-0 24002751 PMC3836491

[B29] Kajikawa A. Zhang L. Lavoy A. Bumgardner S. Klaenhammer T. R. Dean G. A. (2015). Mucosal immunogenicity of genetically modified *Lactobacillus acidophilus* expressing an HIV-1 epitope within the surface layer protein. PLoS ONE 10:e0141713. 10.1371/journal.pone.0141713 26509697 PMC4624987

[B30] Kelly P. Maguire P. B. Bennett M. Fitzgerald D. J. Edwards R. J. Thiede B.. (2005). Correlation of probiotic *Lactobacillus salivarius* growth phase with its cell wall-associated proteome. FEMS Microbiol. Lett. 252, 153–159. 10.1016/j.femsle.2005.08.051 16214296

[B31] Kleerebezem M. Hols P. Bernard E. Rolain T. Zhou M. Siezen R. J.. (2010). The extracellular biology of the lactobacilli. FEMS Microbiol. Rev. 34, 199–230. 10.1111/j.1574-6976.2009.00208.x 20088967

[B32] Kleerebezem M. Vaughan E. E. (2009). Probiotic and gut lactobacilli and bifidobacteria: molecular approaches to study diversity and activity. Annu. Rev. Microbiol. 63, 269–290. 10.1146/annurev.micro.091208.073341 19575569

[B33] Lebeer S. Vanderleyden J. De Keersmaecker S. C. (2008). Genes and molecules of lactobacilli supporting probiotic action. Microbiol. Mol. Biol. Rev. 72, 728–764. 10.1128/MMBR.00017-08 19052326 PMC2593565

[B34] Marco M. L. Pavan S. Kleerebezem M. (2006). Towards understanding molecular modes of probiotic action. Curr. Opin. Biotechnol. 17, 204–210. 10.1016/j.copbio.2006.02.005 16510275

[B35] Nesvizhskii A. I. Keller A. Kolker E. Aebersold R. (2003). A statistical model for identifying proteins by tandem mass spectrometry. Anal. Chem. 75, 4646–4658. 10.1021/ac0341261 14632076

[B36] O'Flaherty S. Klaenhammer T. R. (2016). Multivalent chromosomal expression of the *Clostridium botulinum* Serotype A Neurotoxin Heavy-Chain Antigen and the *Bacillus anthracis* Protective Antigen in *Lactobacillus acidophilus*. Appl. Environ. Microbiol. 82, 6091–6101. 10.1128/AEM.01533-16 27496774 PMC5068166

[B37] Ow S. Y. Salim M. Noirel J. Evans C. Rehman I. Wright P. C. (2009). iTRAQ underestimation in simple and complex mixtures: “the good, the bad and the ugly”. J. Proteome Res. 8, 5347–5355. 10.1021/pr900634c 19754192

[B38] Pfeiler E. A. Azcarate-Peril M. A. Klaenhammer T. R. (2007). Characterization of a novel bile-inducible operon encoding a two-component regulatory system in *Lactobacillus acidophilus*. J. Bacteriol. 189, 4624–4634. 10.1128/JB.00337-07 17449631 PMC1913432

[B39] Pot B. Ludwig W. Kersters K. Schleifer K.-H. (1994). Taxonomy of lactic acid bacteria, in Bacteriocins of Lactic Acid Bacteria: Microbiology, Genetics and Applications, eds De Vuyst L. Vandamme E. J. (Boston, MA: Springer), 13–90.

[B40] Rauniyar N. Yates J. R. (2014). Isobaric labeling-based relative quantification in shotgun proteomics. J. Proteome Res. 13, 5293–5309. 10.1021/pr500880b 25337643 PMC4261935

[B41] Sanders M. E. Klaenhammer T. R. (2001). Invited review: the scientific basis of *Lactobacillus acidophilus* NCFM functionality as a probiotic. J. Dairy Sci. 84, 319–331. 10.3168/jds.S0022-0302(01)74481-5 11233016

[B42] Sleytr U. B. Schuster B. Egelseer E. M. Pum D. (2014). S-layers: principles and applications. FEMS Microbiol. Rev. 38, 823–864. 10.1111/1574-6976.12063 24483139 PMC4232325

[B43] Thompson A. Schäfer J. Kuhn K. Kienle S. Schwarz J. Schmidt G.. (2003). Tandem mass tags: a novel quantification strategy for comparative analysis of complex protein mixtures by MS/MS. Anal. Chem. 75, 1895–1904. 10.1021/ac0262560 12713048

[B44] Ting L. Rad R. Gygi S. P. Haas W. (2011). MS3 eliminates ratio distortion in isobaric multiplexed quantitative proteomics. Nat. Methods 8, 937–940. 10.1038/nmeth.1714 21963607 PMC3205343

[B45] van Baarlen P. Troost F. J. van Hemert S. van der Meer C. de Vos W. M. de Groot P. J.. (2009). Differential NF-kappaB pathways induction by *Lactobacillus plantarum* in the duodenum of healthy humans correlating with immune tolerance. Proc. Natl. Acad. Sci. U.S.A. 106, 2371–2376. 10.1073/pnas.0809919106 19190178 PMC2650163

[B46] Wang G. Xia Y. Cui J. Gu Z. Song Y. Chen Y. Q.. (2014). The roles of moonlighting proteins in bacteria. Curr. Issues Mol. Biol. 16, 15–22. 10.21775/cimb.016.015 23872606

[B47] Warner J. R. Mcintosh K. B. (2009). How common are extraribosomal functions of ribosomal proteins? Mol. Cell 34, 3–11. 10.1016/j.molcel.2009.03.006 19362532 PMC2679180

[B48] Wells J. M. Mercenier A. (2008). Mucosal delivery of therapeutic and prophylactic molecules using lactic acid bacteria. Nat. Rev. Microbiol. 6, 349–362. 10.1038/nrmicro1840 18345021 PMC7096801

